# O059: Introducing an intervention bundle to reduce the incidence of catheter related infections

**DOI:** 10.1186/2047-2994-2-S1-O59

**Published:** 2013-06-20

**Authors:** E Smid, J Wille, S Greeff de, T Hopmans, M Koek

**Affiliations:** 1 CIb/PREZIES, RIVM, Bilthoven, The Netherlands

## Introduction

Literature indicates that the incidence of catheter related infections can be reduced after implementing a so-called intervention bundle. In line with this, an intervention bundle to prevent catheter related infections was introduced in The Netherlands, aiming (1) at a 90% compliance with the bundle and (2) to reduce the incidence of catheter related infections.

## Objectives

To assess whether a 90% compliance with the bundle and/or a reduction in the incidence of catheter related infections in Dutch hospitals to less than three cases of infection per 1000 catheter-days have been established.

## Methods

In 2009 an intervention bundle consisting of six items was introduced. The six items were hand hygiene, precautions during insertion, cleaning the skin, selection insertion site, daily check on indication, and daily check on insertion site. All Dutch hospitals were asked to register the compliance with the intervention bundle together with the incidence of infections for all central venous catheters. Registration took place from January 2009 until December 2012. The intervention bundle is analysed on registration and compliance level.

## Results

64 from the 93 hospitals in The Netherlands registered for participation, 31 hospitals registered infection-data and 27 registered data about the intervention bundle. Registration of the complete bundle increased from 7% in 2009 to 61% in 2012. During these four years compliance with the bundle increased from 76% to 87%. In 2009, 6 out of the 9 registering hospitals (67%) had an incidence of less than three cases of infection per 1000 catheter-days. In 2012, 20 out of 25 registering hospitals (80%) had less than three infections per 1000 catheter-days. Four hospitals in 2012 (16%) had more than five infections per 1000 catheter-days.

## Conclusion

Compliance with the complete bundle increased over the four years to 87%, but the target of 90% is not yet met. In 2012 a higher proportion of the hospitals had an incidence of less than three infections per 1000 catheter-days (not significantly different), but still some hospitals had more than five infections per 1000 catheter-days. We conclude that for both compliance and infection-rates there is still room for improvement.

## Disclosure of interest

None declared.

